# Defect detection of photovoltaic modules based on improved VarifocalNet

**DOI:** 10.1038/s41598-024-66234-3

**Published:** 2024-07-02

**Authors:** Yanfei Jia, Guangda Chen, Liquan Zhao

**Affiliations:** 1 College of Electrical and Information Engineering, Beihua University, Jilin, 132013 China; 2 College of Electrical Engineering, Northeast Electric Power University, Jilin, 132012 China

**Keywords:** Electrical and electronic engineering, Renewable energy, Techniques and instrumentation

## Abstract

Detecting and replacing defective photovoltaic modules is essential as they directly impact power generation efficiency. Many current deep learning-based methods for detecting defects in photovoltaic modules focus solely on either detection speed or accuracy, which limits their practical application. To address this issue, an improved VarifocalNet has been proposed to enhance both the detection speed and accuracy of defective photovoltaic modules. Firstly, a new bottleneck module is designed to replace the first bottleneck module of the last stage convolution group in the backbone. This new module includes both standard convolution and dilated convolution, enabling an increase in network depth and receptive field without reducing the output feature map size. This improvement can help to enhance the accuracy of defect detection for photovoltaic modules. Secondly, another bottleneck module is also designed and used to replace the original bottleneck module used in the fourth stage convolution group of the backbone. This new module has smaller parameters than the original bottleneck module, which is useful to improve the defect detection speed of the photovoltaic module. Thirdly, a feature interactor is designed in the detection head to enhance feature expression in the classification branch. This helps improve detection accuracy. Besides, an improved intersection over union is proposed and introduced into the loss function to measure the difference between the predicted and ground truth boxes. This is useful for improving defect detection accuracy. Compared to other methods, the proposed method has the highest detection accuracy. Additionally, it also has a faster detection speed than other methods except for the DDH-YOLOv5 method and the improved YOLOv7 method.

## Introduction

Solar photovoltaic (PV) energy has gained significant attention and has undergone rapid global development in the past decade. The deployment of PV technology has expanded quickly, including both centralized PV farms and distributed PV generation, thanks to technological advancements and cost reductions. This clean energy source has become increasingly popular due to its benefits in reducing carbon emissions and producing renewable energy. Global renewable-based power capacity is set to rise 50% between 2019 and 2024, with solar photovoltaic accounting for 60% of the increase, according to the International Energy Agency. It is essential to ensure the longevity of photovoltaic modules to promote the development of solar technology, but these PV cells are vulnerable to various external factors. Even minor manufacturing defects can cause damage to the modules. Additionally, during transportation and installation, vibrations and shocks can pose potential risks of breakage. Defects such as the finger, thick_line, corner, and black_core can make PV modules unusable, and microcracks that are hard to observe will potentially affect future output power and lifetime. Defects in PV cells can lead to module failure, which can result in reduced power output and pose safety risks to the system. Therefore, it is essential to conduct regular inspections and maintenance of photovoltaic modules to ensure maximum output from the PV system throughout its lifespan^1^. However, with the rising use of photovoltaic and ongoing installation of large-scale photovoltaic systems worldwide, the manual inspection methods and volt-ampere characteristics of the integrated tester methods do not meet the demand. They have lower detection efficiency and fewer types of defects^2^. Therefore, it is crucial to promptly and accurately detect defects in photovoltaic cells to ensure long-term stable operation of the PV power generation system.

The detection of defects in photovoltaic models can be categorized into two types. The first type involves analyzing the characteristic curves of electrical parameters, such as current, voltage, and power of the photovoltaic system. This analysis is combined with environmental parameters, such as irradiance and temperature, to identify different types of defects. The second method involves analyzing the visible light, electroluminescence(EL) image, or thermal imaging characteristics of photovoltaic arrays to detect different types of defects. Detecting defects in photovoltaic modules through electrical characteristics is expensive due to the costly deployment of sensor equipment and human resources, complex wiring process, lack of system flexibility, difficulty in pinpointing exact fault locations, and high maintenance costs. Electroluminescence (EL) imaging is a widely used technology for detecting defects in photovoltaic (PV) modules due to its high resolution. This non-destructive technology provides exceptional imaging resolution, particularly useful for detecting microcracks. In the EL images, faulty PV modules with cracks and other defects appear as dark gray lines and areas. However, traditional methods that relied on manual features were introduced initially for defect detection in EL images. These methods were limited in their performance, as they heavily relied on extensive manual design experiments. Deep learning methods have become the mainstream method for detecting defects in EL images due to their strong feature-capturing ability.

Deep learning methods train models using a large set of labeled images. The model extracts and learns image features automatically, without human intervention. Deep learning methods are widely used for recognition and classification tasks, such as detecting defects in PV modules. The modern object detection method based on deep learning can be divided into two types: one-stage method^3^ and two-stage method^4^. Improving detection speed is the focus of the one-stage method, while the two-stage method emphasizes detection accuracy. In the practical detection of photovoltaic module defects, we should consider not only the detection speed but also the detection accuracy. The VarifocalNet is an anchor-free detection method and has higher detection accuracy^5^. To further improve both the detection accuracy and speed for detecting photovoltaic module defects, a detection method of photovoltaic module defects in EL images with faster detection speed and higher accuracy is proposed based on VarifocalNet.

The main contributions of our proposed method are as follows:

- The larger receptive field is beneficial to improve the network feature extraction. The larger receptive field is beneficial to improve the network feature extraction. If we increase the perceptual field by increasing the network depth, the feature map size will be reduced. The photovoltaic module defect is relatively smaller, so the smaller feature map will reduce the detection accuracy. To increase the perceptual field without reducing the feature map size, we design a new bottleneck module that consists of standard convolution and dilated convolution. To improve the detection accuracy of photovoltaic module defects based on VarifocalNet, we use the new bottleneck module to replace the first bottleneck module used in the last stage convolution group of backbone in VarifocalNet.
- The bottleneck module of VarifocalNet includes $3\times 3$ convolution, it occupies a higher number of parameters. To reduce the number of parameters for the bottleneck module, we design an improved bottleneck module by using a $3\times 1$ convolution and a $1\times 3$ convolution to replace the $3\times 3$ convolution. The sum of parameters of $3\times 1$ convolution and $1\times 3$ convolution is smaller than the $3\times 3$ convolution. To speed up the detection speed of photovoltaic module defects based on VarifocalNet, we use the proposed bottleneck module to replace the original bottleneck module used in the fourth convolution group of backbone in VarifocalNet.
- The interactor in VarifocalNet directly affects detection accuracy. To improve the detection accuracy of photovoltaic module defects, a new feature interactor is designed and introduced into the detection head. The proposed feature interactor utilizes dynamic convolution to deduce the feature relationships between local and global features within the feature map. This enables the output interactive features to accurately reflect the feature distinctions between different types of objects and also the feature associations between similar objects. In the end, these features are fused to the classification branch to enhance the feature representation of the classification branch.
- An improved regression loss function is proposed to improve the accuracy of detecting defects in photovoltaic modules. The new loss function is based on the position information of the predicted box and is used to construct the complete loss function of the photovoltaic module defect detection network. This will result in better performance and more accurate detection of defects in photovoltaic modules. The improved loss function is better at measuring the offset between predicted and ground truth boxes, resulting in improved detection accuracy.

In summary, to speed up detection speed, we design a bottleneck module with smaller parameters to replace the original bottleneck module used in the fourth convolution group of backbone in VarifocalNet. To improve detection accuracy, we design a bottleneck module without reducing feature map size to replace the first bottleneck module used in the last stage convolution group of backbone in VarifocalNet. To further improve detection accuracy, we also designed a new feature interactor and improved the regression loss function.

## Related work

This section briefly overviews the detection method of photovoltaic module defects based on deep learning. Deep learning is considered a promising machine learning technique and has been adopted in industrial monitoring^6^, object detection^7,8^ and image super-resolution^9^. The deep learning method also has been widely used in photovoltaic module defect detection^10^. To reduce the detection network complexity, Akram et al.^11^ proposed a light convolution neural network based on a visual geometry group network for detecting photovoltaic cell cracking defects. It requires lower computational power, so it can detect defects without using a graphics processing unit. Although it has a faster detection speed, detection accuracy is lower than the methods that are based on normal convolution neural networks. Li et al.^12^ proposed a deep convolution neural network for detecting photovoltaic module defects by using the aerial infrared images obtained from unmanned aerial vehicles. The infrared images taken by unmanned aerial vehicles have limited clarity and defects are smaller in infrared images, so it can only identify larger defects with very distinct features. To improve the accuracy of fault detection in photovoltaic farms, Roberto et al.^13^ proposed to use improved Mask R-CNN to detect the photovoltaic fault and published a newly annotated dataset. Chen et al.^14^ proposed a multi-scale Faster RCNN model for detecting defects in Electroluminescence images of photovoltaic cells. To improve detection accuracy. it designed a path aggregation feature pyramid network to extract more multi-scale high-level semantic features and a region recommendation network based on a convolutional block attention module. It has a higher accuracy of crack defect detection than Faster RCNN. Tao et al.proposed an improved residual module based on deformable convolution and attention module to improve Faster RCNN^15^. It improves the detection performance of small defects of PV modules and boundary box regression accuracy. Tang et al.^16^ developed a generative adversarial network that generates EL images to overcome the limited number of available samples. An efficient network was also proposed to realize defect detection from the generated EL images. Zhao et al.^17^ also designed an EL image generation model based on GAN structure and combined the traditional data expansion method to expand the dataset. Besides, an intelligent algorithm based on the high-resolution network was also proposed for the defect detection of photovoltaic modules. Other EL image generation methods were also proposed^18^. To improve the defects classification and detection results in raw solar cell EL images, Su et al.^19^ proposed a novel complementary attention network and a region proposal attention network, and introduced the proposed networks into the Faster RCNN. To improve the average precision and detection speed of defect detection of PV modules, Su et al.^20^ designed attention module-based top-down and bottom-up architecture to accomplish multi-scale feature fusion. They name the proposed module as Bidirectional Attention Feature Pyramid Network (BAFPN). It can make all layers of the pyramid share similar semantic features. They introduced it into Faster R-CNN to improve the detection speed and accuracy of defect detection of PV modules. To improve the accuracy of photovoltaic module defect detection, Guo et al.^21^proposed an improved mask R-CNN. The spatial attention mechanism and atrous spatial pyramid pooling operation were introduced into the original mask R-CNN. Although the improved mask R-CNN has a lower detection speed, it has higher detection accuracy than the original mask RCNN.To improve the accuracy of hot spot detection in infrared images, Wang et al.^22^ proposed an improved Faster R-CNN. It used the SpotFPN module to replace the FPN module in the Faster R-CNN method and used the data enhancement method to expand the infrared hot spot dataset. To improve the detection accuracy, Chen et al.^23^ proposed an improved abnormal detection method based on Faster R-CNN for the defect detection of PV modules. They introduced a lightweight channel and spatial convolution attention module, a new clustering algorithm, and improved loss function into Faster R-CNN.

To improve the speed of photovoltaic module defect detection, Meng et al.^24^ proposed a YOLO-based object detection algorithm YOLO-PV based on YOLOv4 for detecting photovoltaic module defects in electroluminescence (EL) images. It simplified the backbone and path aggregation network of YOLOv4 to reduce the complexity of the network. The YOLO-PV has better detection performance than YOLOv4. To make the detection method of photovoltaic module defects be employed on edge-cloud computing infrastructure, Tang et al.^25^ proposed cloud-edge architecture and the algorithmic solution across the developed inspection system covering the edge devices. To improve the accuracy of hotspot detection in infrared thermal images, Shen et al.^26^ proposed a Modified U-Net by introducing batch normalization layers and RMSrop. Jiang et al.^27^ proposed a new method for detecting micro-cracks in photovoltaic modules by attention classification and segmentation network. This method can not only realize the task of classification and location but also segment the defect object. The classification subnet introduces a transfer learning and depth supervision mechanism to effectively extract and fuse multi-scale features, which can perform real-time crack detection tasks. The segmentation subnet using an M-shaped structure and attention mechanism can better extract and fuse multi-level features, which perform pixel-level crack detection on solar modules. To improve detection speed without reducing the accuracy of photovoltaic module defects detection, Cao et al.^28^ proposed YOLOv5s-GBC method based on YOLOv5s. The YOLOv5s is a lightweight network of YOLOv5. To further improve detection speed, it used the GhostConv which is a lightweight module to modify the backbone, and used the Gaussian error linear unit activation function to replace the original activation function in YOLOv5s. Besides, to balance the detection accuracy, a bidirectional feature pyramid network and attention mechanism were also introduced into YOLOv5s.To improve the accuracy of photovoltaic module defects detection based on lightweight YOLO, Wang et al.^29^ proposed PV-LOLO based on YOLOX. Zhang et al.^30^ also proposed an improved YOLOv5 to improve the accuracy and speed of the defect detection of PV modules. It incorporated the deformable convolution into cross stage partial module of YOLOv5. It also utilized the Mosaic and MixUp fusion data enhancement, K-meams++ clustering anchor box algorithm, and CIOU loss function to improve the performance of YOLOv5. Mazen et al.^31^ proposed an improved YOLOv5 for an automatic PV defect detection system in EL images. They introduced the global attention module into the traditional YOLOv5 model to improve the accuracy of defect detection of PV modules. Besides, the adaptive feature space fusion and distance intersection over union suppression were also introduced into the YOLOv5. The improved YOLOv5 has higher detection accuracy than YOLOv8. Cao et al.^32^ proposed an improved YOLOv8-GD deep learning model based on YOLOv8s for defect detection in electroluminescence images of solar photovoltaic modules. It has a faster detection speed and higher detection accuracy than the other lightweight methods. Hakan^33^ proposed an improved version of YOLOv7 by introducing the ghost module and global attention mechanism (GAM) into the backbone, which improved the detection speed and accuracy of PV modules for defect detection. Besides, many object detection methods based on deep learning are also can be used in detecting photovoltaic module defects^34,35^.

The overview of defect detection of photovoltaic modules based on deep learning is shown in Table 1. The above defect detection methods of photovoltaic modules based on deep can be divided into three categories that are based on two-stage method, based on one-stage method, and based on GAN and two-stage method. Two-stage method prioritize improving detection accuracy, while one-stage methods focus more on enhancing detection speed. There are rarely improvements in both detection speed and detection at the same time. To improve both detection speed and accuracy, an improved method for detecting photovoltaic module defects is proposed based on VarifocalNet.

**Table 1:** The overview of defect detection of photovoltaic modules based on deep learning.

Category	References	Strengths	Shortcomings
Based on the two-stage method	Li et al.^12^, Roberto et al.^13^, Chen et al.^14^, Tao et al.^15^, Su et al.^19^, etc.	Higher detection accuracy	Slower detection speed
Based on the one-stage method	Meng et al.^24^, Tang et al.^25^, Shen et al.^26^, Jiang et al.^28^, etc.	Faster detection speed	Lower detection accuracy
Based on GAN and the two-stage method	Tang et al.^16^, Zhao et al.^17^, Romero et al.^18^	Higher detection accuracy	Fewer types of defects

## Photovoltaic modules defect detection

### Improved feature extraction network

**Figure 1:**
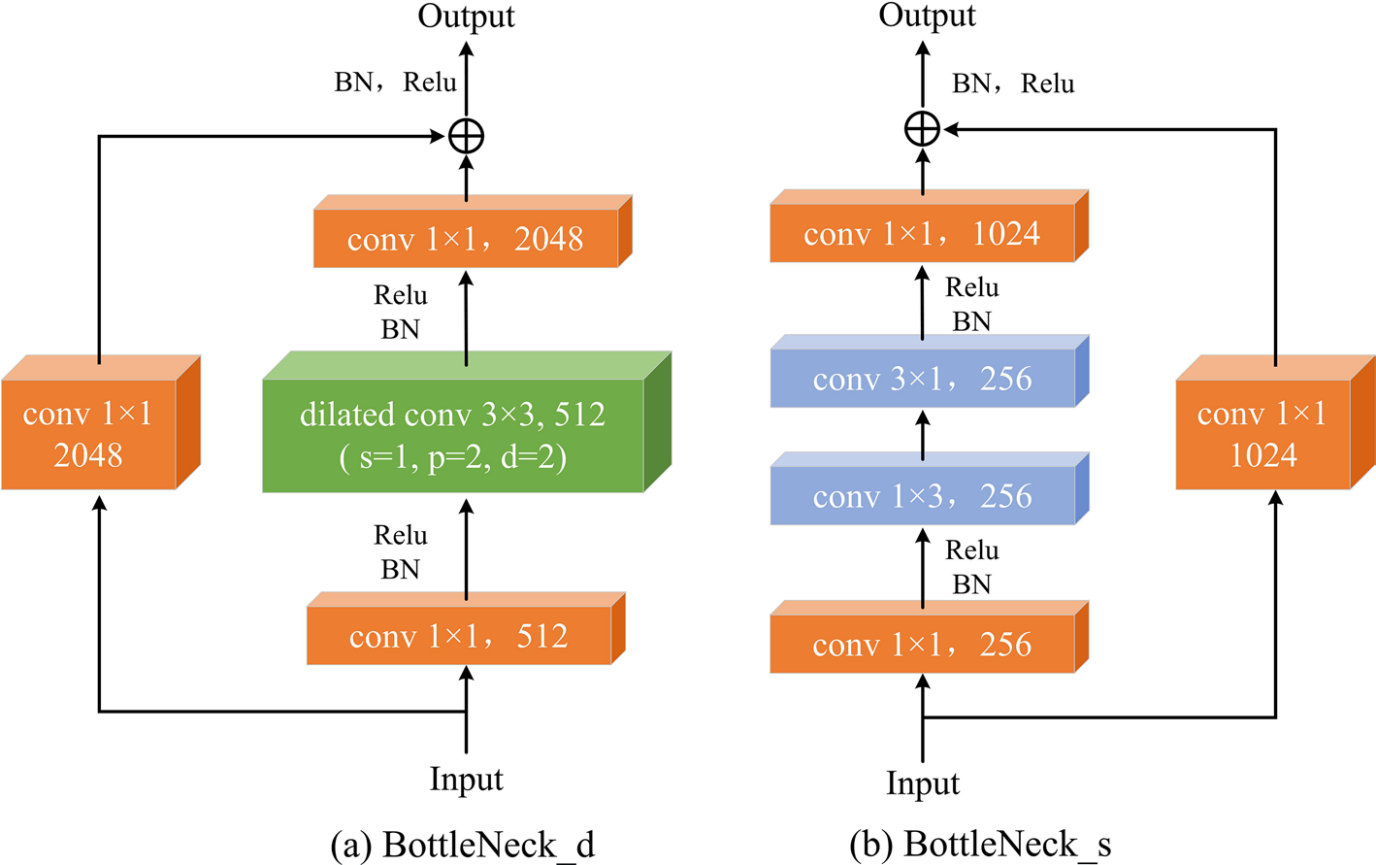
Proposed bottleneck modules.

In VarifocalNet, ResNet-101 is used as the backbone for feature extraction. ResNet-101 comprises five convolutional groups, as shown in the Table 2. The convolutional group is comprised of bottleneck modules. The five convolution groups contain 1, 3, 4, 23, and 3 bottleneck modules, respectively. The output feature map size is reduced to half of the input feature map size for every convolutional group. The last convolutional group generates the smallest output feature map size. However, smaller feature maps are not ideal for detecting smaller objects, such as the relatively smaller photovoltaic module defect. If we use ResNet-101 as the backbone to extract features, the last convolutional group cannot effectively capture photovoltaic module defect features. To solve the problem, we designed a new bottleneck module that is shown in Fig. 1a.

**Table 2:** Original ResNet101 and improved ResNet101.

Original ResNet101	Improved ResNet101
Input image (Width=W, Hight=H, Channel=C=3)	Input image (Width=W, Hight=H, Channel=3)
Group1: Conv2d (kerenel=7, stirde=2), (W/2, H/2, C=64)	Group1: Conv2d (kerenel=7), stirde=2, (W/2, H/2, C=64)
Maxpool (kerenl=3, stride=2), (W/4, H/4, C=64)	Maxpool (kerenl=3, stride=2), (W/4, H/4, C=64)
$$\begin{array}{l} \mathrm{{Group2 :}}\left[ \begin{array}{l} \mathrm{{1 \times 1, 64}} \\ \mathrm{{3 \times 3, 64}} \\ \mathrm{{1 \times 1, 256}} \\ \end{array} \right] \times 3 \\ \mathrm{{ (W/4,H/4,C = 256) }} \\ \end{array}$$	$$\begin{array}{l} \mathrm{{Group2 :}}\left[ \begin{array}{l} \mathrm{{1 \times 1, 64}} \\ \mathrm{{3 \times 3, 64}} \\ \mathrm{{1 \times 1, 256}} \\ \end{array} \right] \times 3 \\ \mathrm{{ (W/4,H/4,C = 256) }} \\ \end{array}$$
$$\begin{array}{l} \mathrm{{Group3}} \mathrm{{:} }\left[ \begin{array}{l} \mathrm{{1 \times 1, 128}} \\ \mathrm{{3 \times 3, 128}} \\ \mathrm{{1 \times 1, 512}} \\ \end{array} \right] \times 4 \\ \mathrm{{ }} \mathrm{{(W/8,H/8,C = 256) }} \\ \end{array}$$	$$\begin{array}{l} \mathrm{{Group3}} \mathrm{{:}}\left[ \begin{array}{l} \mathrm{{1 \times 1, 128}} \\ \mathrm{{3 \times 3, 128}} \\ \mathrm{{1 \times 1, 512}} \\ \end{array} \right] \times 4 \\ \mathrm{{ }} \mathrm{{(W/8,H/8,C = 256) }} \\ \end{array}$$
$$\begin{array}{l} \mathrm{{Group4}} \mathrm{{:}}\left[ \begin{array}{l} \mathrm{{1 \times 1, 256}} \\ \mathrm{{3 \times 3, 256}} \\ \mathrm{{1 \times 1, 1024}} \\ \end{array} \right] \times 23 \\ \mathrm{{(W/16,H/16,C = 512)}} \\ \end{array}$$	$$ \mathrm{{Group4:}}\begin{array}{*{20}c} {BottleNeck\_s \times 23} \\ {(W/16,H/16,C = 1024)} \\ \end{array} $$
$$\begin{array}{l} \mathrm{{Group5}} \mathrm{{:}}\left[ \begin{array}{l} \mathrm{{1 \times 1, 512}} \\ \mathrm{{3 \times 3, 512}} \\ \mathrm{{1 \times 1, 2048}} \\ \end{array} \right] \times 3 \\ \mathrm{{(W/32,H/32,C = 2048) }} \\ \end{array}$$	$$ \mathrm{{Group5:}}\begin{array}{*{20}c} {BottleNeck\_d \times 1} \\ {\left[ \begin{array}{l} 1 \times 1,512 \\ 3 \times 3,512 \\ 1 \times 1,2048 \\ \end{array} \right] \times 2} \\ {(W/16,H/16,C = 2048)} \\ \end{array}$$

We name the bottleneck module that is shown in Fig. 1a BottleNeck_d. It consists of two branches. The first main branch is composed of a $1\times 1$ convolution layer with Batch Normalization (BN) and ReLU activation, followed by a 3x3 dilated convolution with BN and ReLU activation, and another $1\times 1$ convolution layer. This branch is primarily responsible for feature extraction. The second branch contains only a $1\times 1$ convolution, mainly aimed at preserving input features and reducing feature loss. Three 1x1 convolutions are primarily responsible for adjusting the number of feature channels. Dilated convolution plays a major role in feature extraction and enlarging the receptive field. The output features of the two branches are fused by element-wise sum operation and then passed through BN and ReLU activation functions to obtain the final output features of BottleNeck_d. In comparison with the original module, we replace the conventional $3\times 3$ standard convolution with a $3\times 3$ dilated convolution of dilated rate 2 and padding 2. According to (1) for calculating the output feature map size of dilated convolutions, we can determine that the output feature map size of this module remains unchanged compared to the input feature map size. The formula for calculating the output feature map size of dilated convolution is as follows:

1 $$\begin{aligned} H_{out}= & {} \frac{{(H_{in} + 2 \times P - (K - 1) \times d - 1)}}{s} + 1 \end{aligned}$$

2 $$\begin{aligned} W_{out}= & {} \frac{{(W_{in} + 2 \times P - (K - 1) \times d - 1)}}{s} + 1 \end{aligned}$$

where H_out and W_out respectively represent the height and width of the output feature map. The H_out and W_out respectively represent the height and width of the input feature map. The *P* and *s* respectively represent the padding and stride of dilated convolution. The *K* and *d* respectively represent the kernel size and dilated rate of dilated convolution. In BottleNeck_d, the dilated convolution has a kernel size of 3, a stride of 1, a dilation rate of 2, and a padding of 2. Substituting these values into (1) and (2), we find that the output feature map size is equal to the input feature map size. This proves that BottleNeck_d does not change the output feature map size. We use BottleNeck_d to replace the first bottleneck module in the last convolutional group of ResNet-101. The size of the input and output feature maps are the same for the $3\times 3$ dilated convolution, so the size of the input and output feature maps are the same for the proposed BottleNeck_d. Therefore, we use the proposed BottleNeck_d as the first bottleneck module of the last convolution group in ResNet-101 to make the size of the output feature map the same as the size of the input feature map. Compared with the original ResNet-101, we increased the size of the minimum feature map. This can improve the detection accuracy of the smaller defects.

The formula for the receptive field of the single standard convolutional is shown in the following:

3 $$\begin{aligned} RF = k + (k - 1) \end{aligned}$$

where *RF* is the receptive field. *k* is the kernel size of standard convolutional. The receptive field of single dilated convolutional is shown in the following:

4 $$\begin{aligned} RF = k + (k - 1) \times (d - 1) \end{aligned}$$

where *d* is the dilated rate of dilated convolution. Therefore, according to (3) and (4), we can obtain that the receptive field of $3\times 3$ standard convolutional is $3\times 3$ and the receptive field of $3\times 3$ dilated convolutional with the dilated rate of 2 is $5\times 5$. The $3\times 3$ dilated convolution has a larger receptive field than the $3\times 3$ standard convolution, which helps extract more semantic information. The size of the output feature map of the last convolution group is the same as the output feature map of the fourth convolution group, so it can reduce one upsampling operation in feature fusion. This can reduce the loss of fine-grained features and improve feature utilization.

The standard convolution and dilated convolution have the same formulas for calculating the number of parameters. It can be expressed as:

5 $$\begin{aligned} p = c_i \times c_o \times k_w \times k_h \end{aligned}$$

where $$c_i$$ and $$c_o$$ are the number of input channels and output channels for the standard convolution and dilated convolution, respectively. The $$k_w $$ and $$k_h $$ are the width and height of the convolution kernel for standard convolution and dilated convolution, respectively. We just use the $$3\times 3$$ dilated convolution to replace the $$3\times 3$$ standard convolution in the bottleneck module, so they have the same number of input channels and output channels. Therefore, the $$3\times 3$$ dilated convolution and $$3\times 3$$ standard convolution have the same number of parameters. This means that the proposed BottNeck_d does not increase the computation of the network.

To improve the detection speed, we propose the second bottleneck model named BottleNeck_s, shown in Fig. 1b. It also consists of two branches. The first main branch is composed of a $1\times 1$ convolution layer with Batch Normalization (BN) and ReLU activation, followed by a $1\times 3$ convolution, a $3\times 1$ convolution with BN and ReLU activation, and another $1\times 1$ convolution layer. This branch is also primarily responsible for feature extraction. The second branch also contains only a $1\times 1$ convolution, mainly aimed at preserving input features and reducing feature loss. Three $1\times 1$ convolutions are primarily responsible for adjusting the number of feature channels. The $1\times 3$ convolution and $3\times 1$ play a major role in feature extraction. The output features of the two branches are also fused by element-wise sum operation and then passed through BN and ReLU activation functions to obtain the final output features of BottleNeck_s. In comparison with the original module, we use a $1\times 3$ convolution and a $3\times 1$ convolution instead of the original $1\times 3$ convolution in the bottleneck module. In the end, we use the BottleNeck_s to replace all bottleneck modules in the fourth convolution group that has the largest number of bottleneck modules. In the fourth convolution group, the number of input channels and output channels is 256. Therefore, according to (5) without considering bias, in the fourth convolution group, the number of parameters for the original $3\times 3$ convolution is:

6 $$\begin{aligned} p_s = 3 \times 3 \times 256 \times 256 = \mathrm{{589824}} \end{aligned}$$

According to (5) without considering bias, in the fourth convolution group, the sum number of parameters for $1\times 3$ convolution and $3\times 1$ convolution is:

7 $$\begin{aligned} p_d = \mathrm{{256}} \times \mathrm{{256}} \times \mathrm{{1}} \times \mathrm{{3 + 256}} \times \mathrm{{256}} \times \mathrm{{3}} \times \mathrm{{1}} = \mathrm{{393216}} \end{aligned}$$

According to (6) and (7), the sum number of parameters for $1\times 3$ convolution and $3\times 1$ convolution is reduced by 32.3% than the original $3\times 3$ convolution. According to (5) the sum number of parameters for the original fourth convolution group and improved fourth convolution group are 25493504 and 20971520, respectively. The number of parameters for the improved fourth convolution group reduces by 17.74% than the original fourth convolution group.

The improved ResNet101 is shown in Table 1. Compared to the original model, we replaced all bottleneck modules in the fourth group convolution with BottleNeck_s, and we substituted the first bottleneck module in the fifth group convolution with BottleNeck_d. Based on the above analysis, BottleNeck_s has relatively fewer computations. Since the fourth group module contains the largest number of bottleneck modules, replacing all modules with BottleNeck_s effectively reduces computation. The first bottleneck module in the fifth group module determines the output feature map size of the entire group module. By using BottleNeck_d to replace the first bottleneck module, the output feature map maintains the same size as the input feature map, thus keeping the feature map size unchanged. It can reduce one upsampling operation in feature fusion. This can reduce the loss of fine-grained features and improve feature utilization.

### Improved detection head

**Figure 2:**
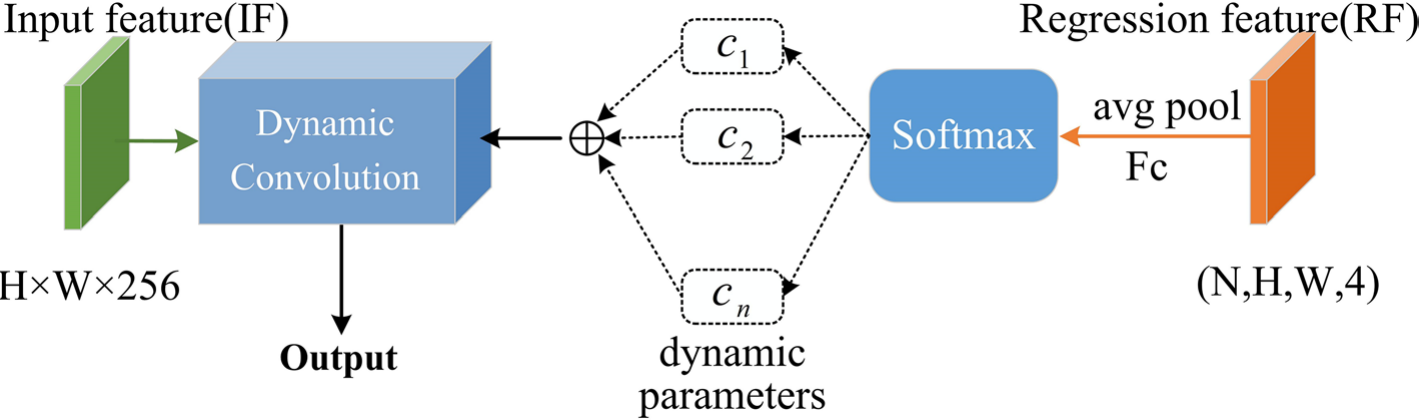
Proposed feature interactor.

In the detection head of the VarifocalNet model, the role of the regression branch is to generate bounding boxes based on regression features. Although the selected targets’ positions vary, targets of the same category still exhibit similar features in different positions. There are also feature differences between targets and backgrounds, as well as among different categories of targets. Therefore, the regression branch contains features that are relevant to categories and can be utilized by the classification task. How to extract potential category-related features from the regression branch and use them for the classification task is crucial for further improving detector performance. To address this, we propose a feature interactor that operates between the classification branch and the regression branch. The feature interactor can interact with local features and global features through dynamic convolution. Dynamic convolution can be regarded as a variable-parameter convolution with attention, which aggregates multiple convolution kernels, each with the same size. The number of convolution kernels is related to the input volume, so the dynamic nature of dynamic convolution is reflected in the convolution parameters changing with input variations. The structure and workflow of the feature interactor are illustrated in Fig. 2.

The input of the feature interactor comprises multi-level features IF from the feature pyramid output and regression features RF from the regression branch. Pyramid features represent global features of the entire feature map. Regression features originate from the features of N predicted boxes generated by the regression branch. These predicted boxes contain targets of multiple categories located at different positions, making the regression features a local representation of the features. Regression features are used to provide dynamic parameters for the dynamic convolution layer. Initially, global average pooling is applied to compute the average value of pixels, extracting and compressing the regression features. Since the pooling window does not contain weight parameters, this feature processing does not introduce additional parameters. Subsequently, the compressed regression features are mapped to the input of the Softmax function through fully connected layers. The fully connected layers enable the gradients in the classification branch to backpropagate to the regression branch and the feature extraction network during training. The Softmax function assigns normalized attention weights to all convolution kernels. These attention weights are utilized for kernel aggregation, and the aggregated parameters are used to construct the dynamic convolution layer. The process of kernel aggregation and dynamic convolution is depicted as follows:

8 $$\begin{aligned} w= & {} \sum \limits _{n = 1}^N {c_n w_n }, \end{aligned}$$

9 $$\begin{aligned} b= & {} \sum \limits _{n = 1}^N {c_n b_n } \end{aligned}$$

10 $$\begin{aligned} y= & {} g(w^T x + b) \end{aligned}$$

where *y* represents the output feature, $c_n$ denotes the attention weights, and *N* stands for the number of convolution kernels, which is equal to the number of predicted boxes. $w_n$ represents the weight matrix of the *n*-th convolution kernel, $b_n$ represents the bias of the *n*-th convolution kernel, *x* denotes the output feature of the feature pyramid, and *g* represents the ReLU activation function. Aggregating all convolution kernel parameters according to the attention weights yields the aggregated weights *w* and aggregated bias *b*, thus completing the construction of the dynamic convolution layer. Pyramid features are compressed by RoI Align pooling to capture overall spatial features, and the compressed global features are then input into the dynamic convolution layer. The pyramid features undergo dynamic convolution to obtain the final output of the dynamic convolution layer, namely the interactive feature *y*. Finally, the interactive feature is introduced into the classification branch, enabling the classification branch to acquire richer category features. By deriving dynamic parameters from local features and constructing dynamic convolutional layers, dynamic convolution is applied to the global pyramid features, facilitating interaction between global and local features. This interaction mechanism can infer potential feature correlations among objects of the same class, as well as feature differences between objects of different classes and between objects and backgrounds. The feature interactor operating between the regression and classification branches can infer category-related interaction features from pyramid features and regression features using dynamic convolution.

The interactivity of the feature interactor is manifested in two aspects: first, the interaction between local features and global features. During forward propagation, interactive features enhance the richness of category features, thereby improving the model’s classification capability. Secondly, there is an interactor between the classification and regression tasks, where the classification branch can supervise the learning of the regression branch through backpropagation, thereby enhancing the accuracy of bounding box localization. Compared to merely extracting category features through traditional convolutional layers, utilizing a feature interaction in the detection head aids the classification branch in distinguishing between background and target positions, identifying feature correlations among objects of the same class, and discerning feature differences among objects of different classes. The interactive features contain a lot of information related to classifications, which is conducive to improving the richness of classification features in the classification branch.

**Figure 3:**
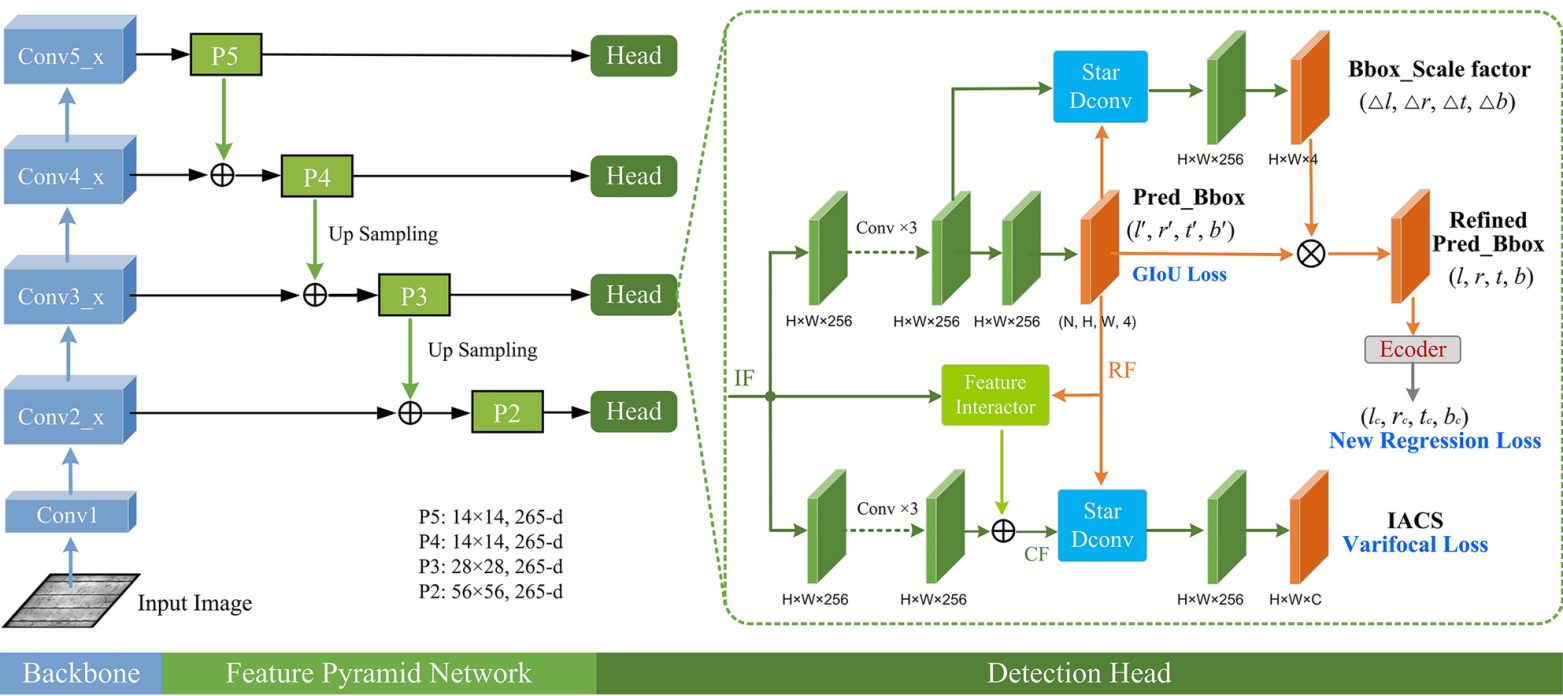
The structure of the improved VarifocalNet.

The structure of the improved VarifocalNet is shown in Fig. 3. It comprises a backbone network (improved ResNet-101) and a feature pyramid network, where the feature pyramid network is utilized to fuse features extracted from various levels of ResNet-101. The detection head consists of a bounding box optimization branch, a bounding box scaling branch, and a classification branch. The bounding box optimization branch learns the correction factors $(\Delta l, \Delta r, \Delta t, \Delta b)$ for the bounding boxes. The regression branch generates initial bounding boxes $(l', r', t', b')$, where $l'$, $r'$, $t'$, $b'$ represent the distances from the key points of the initial bounding boxes to the left, right, top, and bottom boundaries of the bounding boxes, respectively. Finally, the optimized bounding boxes (*l*, *r*, *t*, *b*) are obtained based on the correction factors, where $(l, r, t, b) = (l' \times \Delta l, r' \times \Delta r, t' \times \Delta t, b' \times \Delta b)$.

### Improved regression loss

The original VarifocalNet uses the GIoU loss as a regression loss. The GIoU is expressed as:

11 $$ L_{{GIoU}}  = 1 - \frac{{{\text{A}} \cap B}}{{A \cup B}}{\text{ + }}\frac{{\left| {C - (A \cup B)} \right|}}{{\left| C \right|}} $$

Where B and A represent the predicted box and the ground truth box, while C denotes the minimum enclosing box encompassing both the ground truth box and the predicted box. When one of the bounding boxes of A and B is completely contained by the other, $\frac{{\left| {C - (A \cup B)} \right| }}{{\left| C \right| }} = 0$, and the $\frac{{{\text{A}} \cap B}}{{A \cup B}}$is always a constant value, regardless of how the surrounding box moves in the nearby area. In this case, the GIoU loss cannot measure the degree of deviation between the ground truth box and the predicted box for different surrounding boxes. To overcome the problem, we propose an improved regression loss based on GIoU loss by introducing the center point deviation between the ground-truth box and the predicted box.

The improved GIoU loss is expressed as:

12 $$\begin{aligned} L_{New} = 1 - \frac{{A \cap B}}{{A \cup B}}\mathrm{{ + }}\frac{{\left| {C - (A \cup B)} \right| }}{{\left| C \right| }} + \frac{{16}}{{\pi ^2 }}\left[ \left( \frac{\pi }{4} - \arctan \frac{{l_c }}{{r_c }}\right) ^2 + \left( \frac{\pi }{4} - \arctan \frac{{t_c }}{{b_c }}\right) ^2 \right] \end{aligned}$$

**Figure 4:**
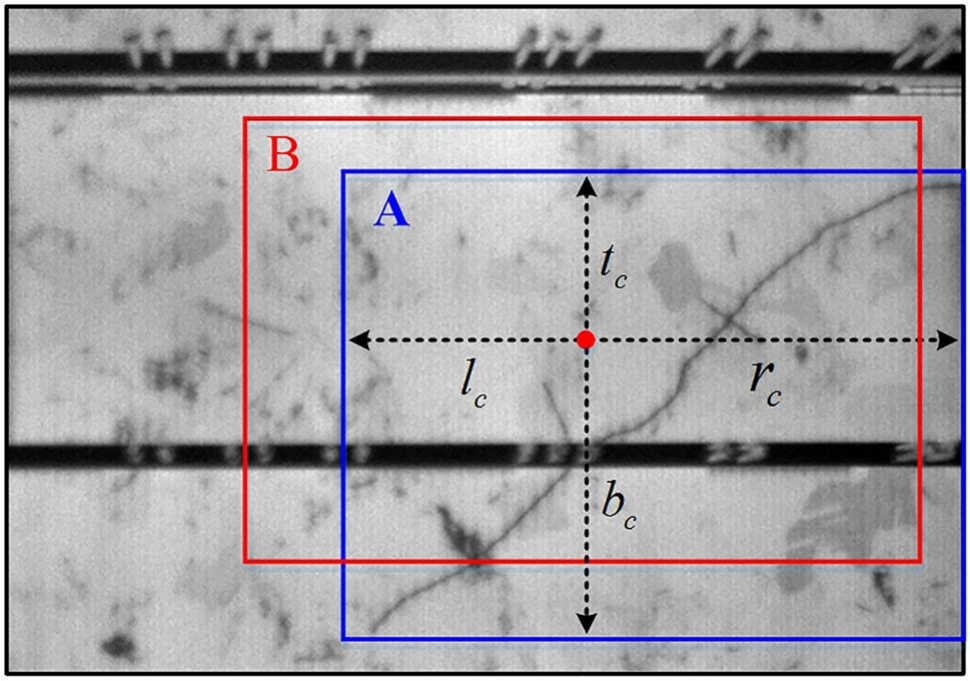
Ground-truth box (A box) and predicted box (B box).

Where, $$l_c$$, $$r_c$$, $$t_c$$, and $$b_c$$ represent the distances from the center of the predicted box to the left, right, top, and bottom boundaries of the ground truth box, respectively. In Fig. 4, B and A correspond to the predicted box and ground truth box, respectively. As the predicted box approaches the ground truth box, the disparities between the $$l_c$$, $$r_c$$, $$t_c$$, and $$b_c$$ become progressively smaller. When the predicted box precisely aligns with the ground truth box, the $$l_c = r_c $$, $$t_c = b_c$$, $$A \cap B\mathrm{{ = }}A \cup B$$ and the $$\left| {C - (A \cup B)} \right| = 0$$. Therefore, the improved GIoU equals zero in the ideal case. When one of the bounding boxes A is completely enclosed by a bounding box B, the probability of the centers of the two boxes coinciding exactly is very small, so the $$l_c /r_c $$ and $$t_c /b_c $$ are different for different predicted boxes in most cases. The surrounding boxes with different positions have different values of loss. Hence, the enhanced Generalized Intersection over Union (GIoU) loss proves to be more effective in quantifying the extent of deviation between the ground-truth box and the predicted box in comparison to the original GIoU loss.

The complete loss based on improved GIoU loss is expressed as:

13 $$\begin{aligned} Loss = \frac{1}{N}\sum \limits _i {\sum \limits _c {VFL(p_{c,i},q_{c,i} )} } + \frac{{\lambda _0 }}{N}\sum \limits _i {q_{c^*,i} L_{GIoU} (bboxx_i },bbox_i^* ) + \frac{{\lambda _1 }}{N}\sum \limits _i {q_{c^*,i} L_{New} (bbox_i,bbox_i^* )} \end{aligned}$$

Where *VFL*() is the varifocal loss that is used in VarifocalNet. $$L_{GIoU}()$$ and $$L_{New} ()$$ are the GIoU loss and our proposed regression loss, respectively. N represents the count of foreground points, while $$\lambda _0$$ and $$ \lambda _1$$ denote the balance weights. $$p_{c,i}$$ and $$q_{c,i}$$ are the predicted and object IoU-aware classification scores for the class *c* at the location *i* on each level feature map of the feature pyramid network, respectively. $$ bbox^*$$, $$bbox_i$$, and $$bboxx_i$$ represent the ground truth box, predicted box, and initial box, respectively.

## Simulation and discussion

**Figure 5:**
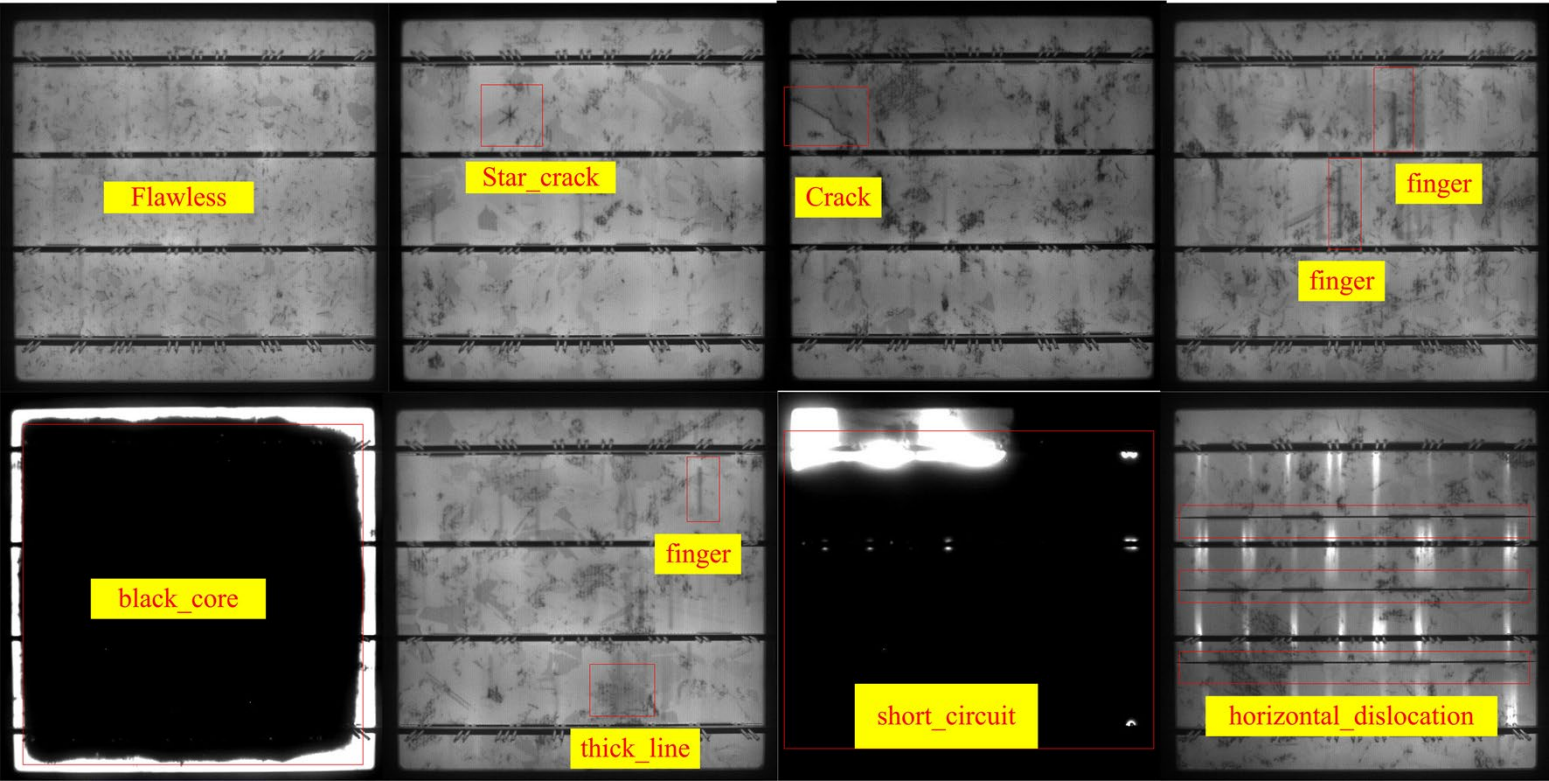
Images without defects and images with defects.

This paper uses the PVEL-AD dataset^36^ to train and test different photovoltaic module defect detection methods. The PVEL-AD dataset comprises over 4,0000 near-infrared images featuring a range of internal defects and diverse backgrounds in photovoltaic modules. The photovoltaic module defects within the PVEL-AD dataset encompass crack,thick_line, black_core, finger_scratch, star_crack, horizontal_dislocation, and short circuit, etc. This paper mainly uses mAP(mean average precision), recall to measure the accuracy, and FPS (frame per second) to measure the detection speed. We use Ubuntu 18.04 as the operating system and PyTorch as the deep learning framework. We use the AdamW to optimize the loss function. The default initial learning rate is 0.0001. The training batch size is 4. We also employ metrics: Precision, Recall, and FPS to measure the performance of defect detection. The Precision and Recall are expressed as:

14 $$\begin{aligned} Precison= & {} \frac{{TP}}{{TP + FP}} \end{aligned}$$

15 $$\begin{aligned} Recall= & {} \frac{{TP}}{{TP + FN}} \end{aligned}$$

where the true positives(TP) is the number of correctly predicted positive samples, False Positives(FP) is the number of incorrectly predicted positive samples and true negatives (TN) is the number of correctly predicted negative samples. The purpose of Precision in defect detection is to measure the accuracy of the model in identifying defects. Specifically, Precision quantifies how many of the detected defects are true defects. High Precision means that the model has a high accuracy in identifying defects, indicating that most of the detected defects are genuine. This is crucial for ensuring the quality of PV modules and the reliability of the detection system. The role of Recall in defect detection is to measure the model’s ability to identify all true defects. The Recall metric quantifies the proportion of true defects successfully identified by the model among all actual defects. A higher recall means that the model has a stronger ability to detect defect samples. The mean Average Precision (mAP) is the mean of the Average Precision (AP) across all categories. Frames Per Second (FPS) is used to measure the number of detected images in one second. A large FPS indicates the method has faster detection. Therefore, we use the mAP and Recall to measure the detection performance of the defect detection model for PV modules, and FPS to measure detection speed. An ideal defect detection model should have high mAP, Recall, and FPS, indicating high detection accuracy and speed. The images without defects and images with defects are shown in Fig. 5.

### Qualitative analysis

**Figure 6:**
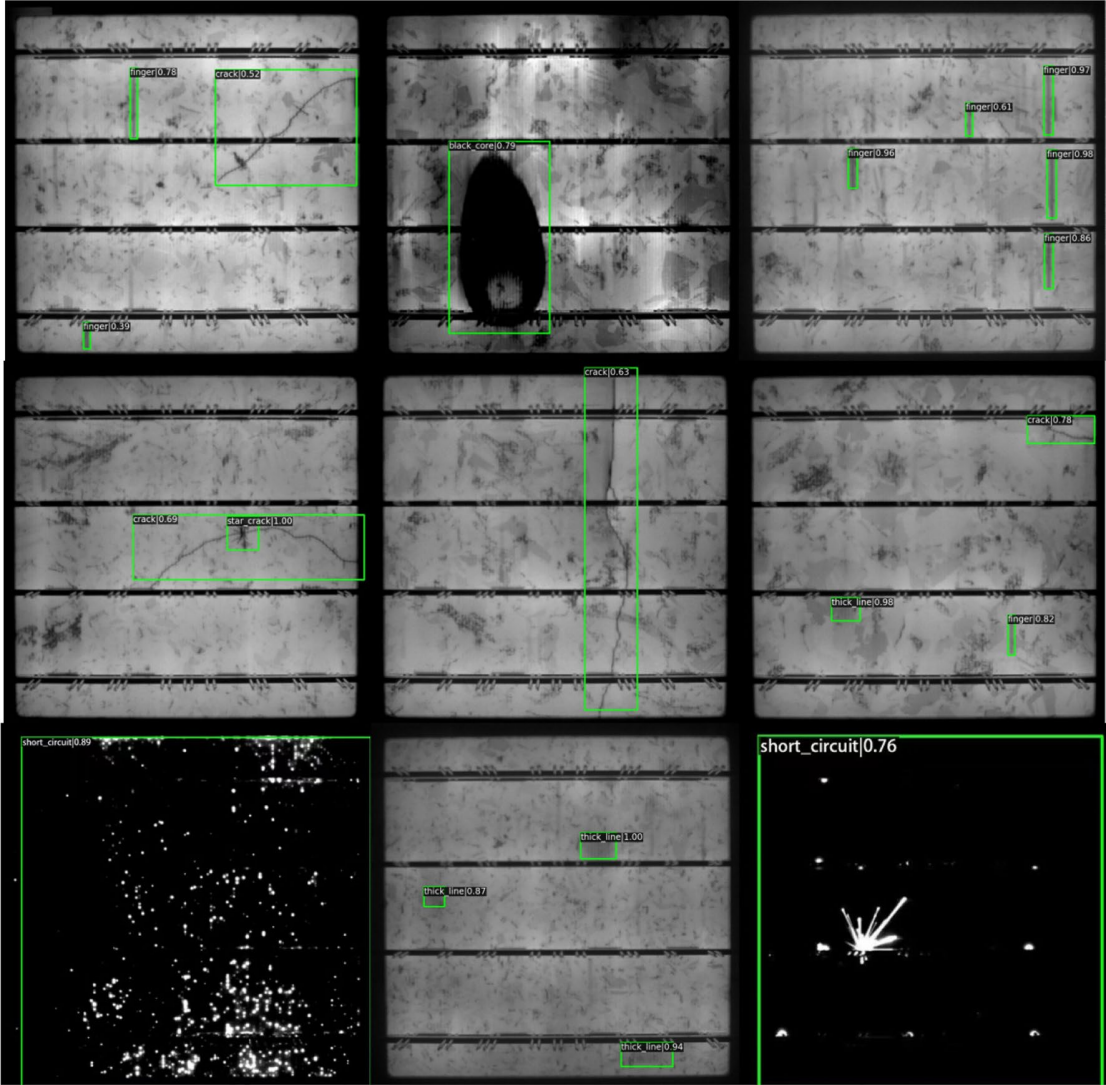
Photovoltaic module detection using the proposed method.

**Figure 7:**
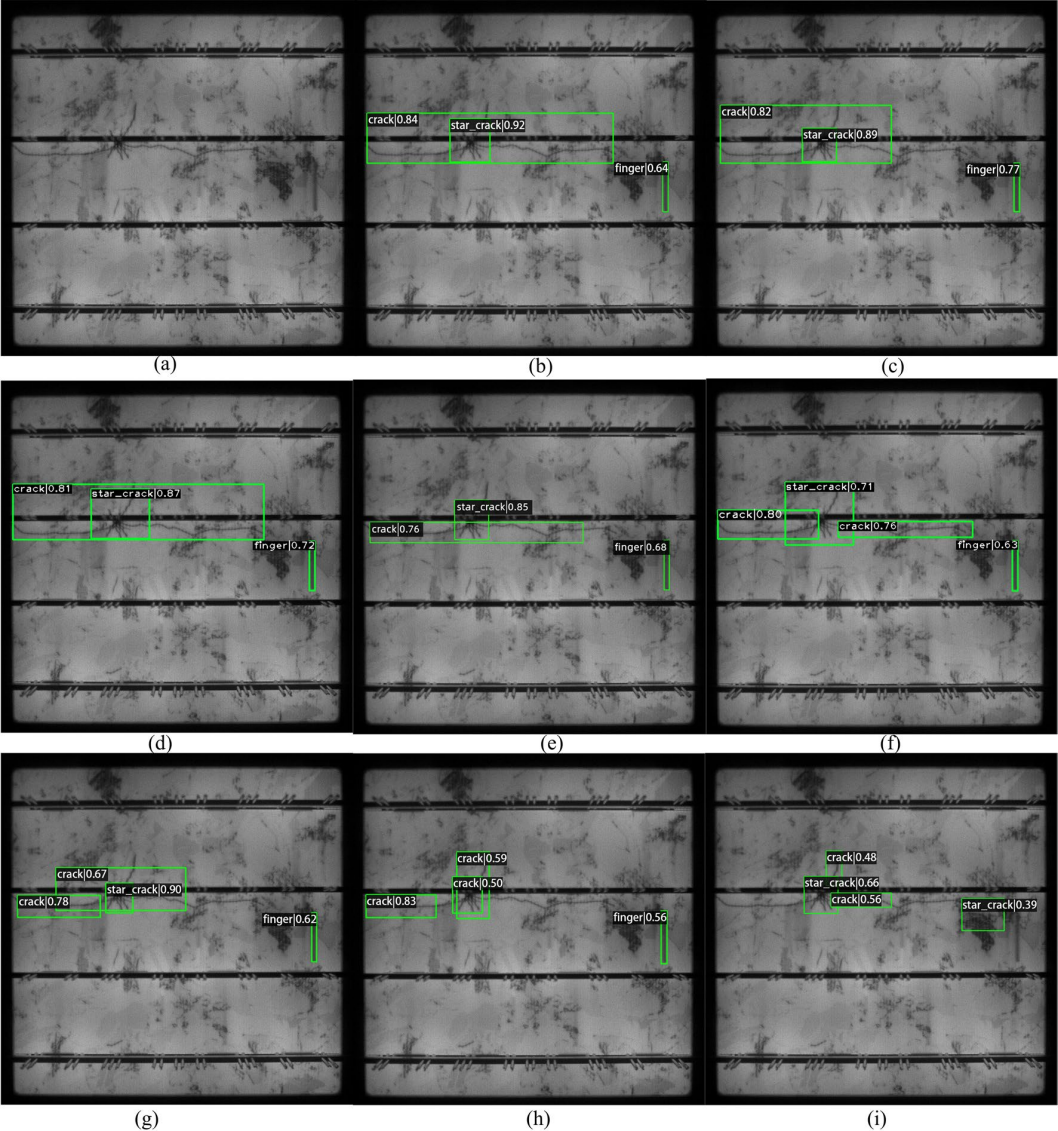
The detection results of different methods for photovoltaic module defect. (**a**) Original image, (**b**) Our method, (**c**) Orignal VarifocalNet method, (**d**) Improved faster R-CNN method, (**e**) Faster GG R-CNN method, (**f**) Improved YOLOv7 method, (**g**) Cascade R-CNN method, (**h**) DDH-YOLOv5 method, (**i**) RetinaNet method.

Firstly, we randomly select nine photovoltaic module images containing defect objects from the test dataset and use the improved VarifocalNet algorithm to detect defects to test the method’s validity. The results are shown in Fig. 6. The proposed method can successfully detect large defect objects such as black core and short circuit defects and small objects, such as thick line and finger scratch defects. Secondly, we select an image containing three different defects that are crack defect, star_crack defect, and finger defect, from the test dataset as a test image. We use RetinaNet^37^, DDH-YOLOv5^35^, Faster GG R-CNN^38^, Cascade R-CNN^4^, VarifocalNet^5^, Improved Faster R-CNN^23^, improved YOLOv7^33^, and our proposed improved VarifocalNet to detect the defects from the selected image. The detection results are shown in Fig. 7. Our method, the VarifocalNet method, improved faster R-CNN and Faster GG R-CNN to successfully detect all defects. The improved YOLOv7, cascade R-CNN, and DDH-YOLOv5 also detect all defects, but the position of the detection boxes is inaccurate for the crack defect. They recognize the largest crack defect as two smaller crack defects. The RetinaNet mistakenly detected a star_crack defect and missed a finger defect. The position of the predicted boxes is the worst. Based on the analysis of Fig. 7, Our method, the VarifocalNet method, improved faster R-CNN, and Faster GG R-CNN have better performance in detection accuracy than others.

### Quantitative analysis

Firstly, we test the performances of our proposed modules and loss function. In this paper, we design an improved feature extraction network based on ResNet-101, an improved detection header, and an improved loss function. We use the improved backbone network, detection head, and loss function to replace the corresponding modules and loss function of the VarifocalNet method to test each proposed component’s effectiveness, respectively. The performances of the VarifocalNet method, VarifocalNet with improved backbone, VarifocalNet with improved detection head, and VarifocalNet with improved loss function are shown in Table 3. The values of mean Average Precision (mAP) of VarifocalNet, VarifocalNet with improved feature extraction network, VarifocalNet with improved detection head, and VarifocalNet with improved loss function are 82.6%, 83.1%, 84.3%, and 83.7%, respectively. Our method has the smallest mAP than others. The recall values of VarifocalNet, VarifocalNet with improved backbone, VarifocalNet with improved detection head, and VarifocalNet with improved loss function are 91.6%, 92%, 93.1%, and 92.4%, respectively. The original VarifocalNet still has the smallest mAP than others. These data show that our improvements are effective. The values of Frames Per Second (FPS) are 13.7, 15.6, 13.1, and 13.4, respectively. The VarifocalNet with an improved backbone has the largest FPS, followed by the original VarifocalNet. These show that our improved feature extraction network can improve the detection speed.

**Table 3:** Performances of VarifocalNet and VarifocalNet with improved modules and loss function.

Methods	mAP(%)	Recall(%)	FPS
VarifocalNet	82.6	91.6	13.7
VarifocalNet with improved feature extraction network	83.1	92	15.6
VarifocalNet with improved detection head	84.3	93.1	13.1
VarifocalNet with improved loss function	83.7	92.4	13.4

Secondly, we compare our complete proposed method with RetinaNet, DDH-YOLOv5, improved YOLOv7, Cascade R-CNN, Faster GG R-CNN, VarifocalNet, and Improved faster R-CNN on the test dataset. The detection accuracy and detection speed are shown in Table 4 for different photovoltaic module defect detection methods. The values of mean Average Precision (mAP) for RetinaNet, DDH-YOLOv5, improved YOLOv7, Cascade R-CNN, Faster GG R-CNN, VarifocalNet, Improved faster R-CNN, and our method are 69.3%, 72.2%, 75.8%, 76.1%, 77.3%, 82.6%, 83.1% and 85.7%, respectively. The values of recall for RetinaNet, DDH-YOLOv5, improved YOLOv7, Cascade R-CNN, Faster GG R-CNN, VarifocalNet, and Improved faster R-CNN, and our method are 77.6%, 83.1%, 89.6%, 90.4%, 91.0%, 91.6%, 92.4% and 94.2%, respectively. The values of Frames Per Second (FPS) for RetinaNet, DDH-YOLOv5, improved YOLOv7, Cascade R-CNN, Faster GG R-CNN, VarifocalNet, Improved faster R-CNN, and our method are 13.8, 16.5, 20.3, 8.1, 10.1, 13.7, 11.3 and 14.9, respectively. Based on the above analysis, our method exhibits the highest mAP and Recall, indicating that the defect detection accuracy of our method is higher than that of other methods. The improved YOLOv7 has the largest FPS, indicating that the defect detection speed of improved YOLOv7 is faster than that of other methods. Our method, improved Faster R-CNN, Faster GG R-CNN, VarifocalNet, and Cascade R-CNN belong to the two-stage method. The DDH-YOLOv5 and improved YOLOv7 belong to the one-stage method. Two-stage Method features a complex network structure, resulting in higher detection accuracy and slower detection speed, whereas the one-stage method employs a relatively simpler network structure, leading to faster detection speed and lower detection accuracy. Although the improved YOLOv7 and DDH-YOLOv5 method has faster detection speed than our method, our method has a higher detection speed than others.

**Table 4:** Performance of different methods.

Method	mAP(%)	Recall(%)	FPS
RetinaNet method	69.3	77.6	13.8
DDH-YOLOv5 method	72.2	83.1	16.5
Improved YOLOv7 method	75.8	89.6	20.3
Cascade R-CNN method	76.1	90.4	8.1
Faster GG R-CNN method	77.3	91.0	10.1
Original VarifocalNet method	82.6	91.6	13.7
Improved faster R-CNN method	83.1	92.4	11.3
Our method	85.7	94.2	14.9

## Conclusions

In this paper, we present two enhanced bottleneck modules that improve the accuracy and speed of object detection. The first module is designed to enhance detection accuracy, while the second is tailored to improve detection speed. We replaced the bottlenecks in the last and fourth stage convolution group of ResNet-101 with the two improved bottlenecks. Additionally, we introduced an interactor to the detection head to further improve detection accuracy. Finally, we proposed an improved GIoU loss and integrated it into the loss function of VarifocalNet to better measure the deviation between the predicted box and the ground truth box.

We test the different methods for detecting the defective photovoltaic module in the PVEL-AD dataset. Initially, we employ the VarifocalNet method as a baseline to assess the efficacy of our distinctively designed modules. The detection accuracy, The VarifocalNet method with our designed detection head has the highest mean detection accuracy, followed by the VarifocalNet method with our improved loss function and the VarifocalNet method with our designed feature extraction network. Regarding detection speed, our designed feature extraction network in combination with the VarifocalNet method exhibits the swiftest detection speed, with the original VarifocalNet and the VarifocalNet method employing our improved loss function following in sequence. Our designed feature extraction network can improve detection accuracy and speed. While our designed detection head and enhanced loss function contribute to improved detection accuracy, it is important to note that they are associated with a reduction in detection speed.

Secondly, we use our completely improved VarifocalNet to compare with other methods. In the defective photovoltaic module detection accuracy, our improved VarifocalNet method has the highest detection accuracy, followed by the improved Faster R-CNN method and the original VarifocalNet method. In the detection speed, the improved YOLOv7 has the fastest detection speed, followed by our DDH-YOLOv5 and Our method. Although our method exhibits a reduction in detection speed, it has higher detection accuracy. In summary, our proposed method stands out with the highest detection accuracy compared to other methods and maintains a detection speed superior to most others, second only to improved YOLOv7 and DDH-YOLOv5. It improves both the detection accuracy and speed for detecting defective photovoltaic modules.

As the distribution of defect targets in photovoltaic modules varies in shape and location, the horizontal prediction boxes corresponding to a non-horizontally distributed target tend to contain more background features. This, in turn, results in the object features containing more feature noise, ultimately limiting the detector’s classification capability. To address this issue, we propose replacing the right-angle horizontal prediction boxes with polar coordinate rotated prediction boxes in our future work.

## Data Availability

The dataset used in this article is publicly available and can be accessed at https://github.com/binyisu/PVEL-AD/.
